# Emotional Intelligence Mitigates the Effects of Customer Incivility on Surface Acting and Exhaustion in Service Occupations: A Moderated Mediation Model

**DOI:** 10.3389/fpsyg.2020.506085

**Published:** 2021-01-21

**Authors:** Dorota Daniela Szczygiel, Róz·a Bazińska

**Affiliations:** Department of Psychology in Sopot, SWPS University of Social Sciences and Humanities, Sopot, Poland

**Keywords:** customer service, customer mistreatment, burnout, emotional labor, emotional skills, trait affectivity

## Abstract

This study contributes to the constantly accumulating evidence on the effects of customer incivility (CI) on service employee exhaustion. Previous research has demonstrated that surface acting (SA) acts as a mediating variable in the relationship between CI and exhaustion. This study extended prior findings in two ways. The results of Study 1 (315 retail sales employees, 62.2% female) demonstrated that SA mediates the positive relationship between CI and exhaustion while controlling for employees’ trait positive and negative affectivity (NA). The results of Study 2 (292 customer service representatives, 51% female) supported a moderated mediation model demonstrating that trait emotional intelligence (EI) buffers the direct and indirect (through SA) effects of CI on exhaustion. Specifically, it was found that employees exposed to many uncivil customer behaviors but high in trait EI reported using less SA and, thus, experienced fewer exhaustion symptoms than their low in trait EI counterparts. These results highlight EI as an important personal resource that mitigates the adverse effects of CI on service employees’ exhaustion, and suggest that organizations should consider implementing EI training programmes for their frontline service employees.

## Introduction

It has been widely recognized that exposure to stressful working conditions can lead to a variety of negative consequences, such as psychological distress, physical illness and mental disorders (American Psychological Association, 2018). One of the adverse effects of work stressors is job burnout, defined as a specific kind of occupational stress that occurs in response to prolonged exposure to job stressors when sufficient resources to compensate for their negative effects are not available (Maslach et al., 2001; Alarcon, 2011). The majority of research on burnout has been conducted among service sector workers who, due to the interpersonal nature of their work, have been described as being particularly susceptible to burnout (Maslach et al., 2001). Another reason for the interest in burnout among service workers is that, in most economically advanced countries, service workers are now the largest occupational group (Wirtz et al., 2015). In the United States, for example, the share of the service sector in employment exceeded 78% in 2018 (World Bank Group, 2019). The growing role of service workers is also noticeable in Poland, a country that has been undergoing socio-economic transformation from a centrally planned system to a market system in recent decades (Gomułka, 2016). As the Polish economy becomes increasingly service-oriented, significant changes in the employment structure are also becoming clearly visible. The share of the service sector in employment increased from 38% in 1991 to over 58% in 2018 (World Bank Group, 2019). The rapidly growing number of service workers in Poland indicates the need for research on the determinants of job burnout in this occupational group.

The basic characteristics of service sector occupations are that employees have to interact with customers on a daily basis (Dormann and Zapf, 2004). On the one hand, interacting with customers can be a source of employee satisfaction, especially for those who enjoy intensive face-to-face social interaction and are guided by prosocial motives and values (Maneotis et al., 2014). On the other hand, however, there is evidence to suggest that dealing with customers can act as a stressor, and there are at least two reasons for this. First, service providers are often exposed to customer mistreatment, described as the “low-quality interpersonal treatment that employees receive from their customers during service interactions” (Koopmann et al., 2015, p. 34). Customer mistreatment can take various forms, ranging from less intense but more frequent, such as ignoring or disrespecting employees, to relatively less frequent but more intense, such as verbal or even physical aggression (Andersson and Pearson, 1999; Dormann and Zapf, 2004; Grandey et al., 2007; Skarlicki et al., 2008; Hershcovis, 2011; Sommovigo et al., 2019b).

The second reason why interacting with customers is considered stressful stems from pressure to satisfy organizational requirements expecting frontline service employees to behave “properly” when dealing with customers who are “always right.” The slogan “the customer is king” defines the work of many service employees nowadays, who are expected to behave courteously and politely toward customers, regardless of the latter’s behavior (Ben-Zur and Yagil, 2005). This means that, in most service contexts, customer service representatives are expected to express positive emotions and suppress negative ones, even in the face of rude and disrespectful customer treatment (Diefendorff and Richard, 2003; Ben-Zur and Yagil, 2005; Goldberg and Grandey, 2007; Diefendorff and Croyle, 2008). In order to comply with organizational requirements, in many situations, employees may need to make an effort to manage affective displays while dealing with customers; this means that they engage in emotional labor through surface acting (SA; i.e., changing their outward emotional display) and/or deep acting (DA; i.e., changing their inner feelings; Hochschild, 1983; Grandey, 2000).

Previous research has demonstrated that employee exhaustion is affected by both customer incivility (CI; for a review, see Sommovigo et al., 2019b) and SA (Hülsheger and Schewe, 2011; Huppertz et al., 2020), with SA mediating the relationship between CI and exhaustion (e.g., Sliter et al., 2010; Adams and Webster, 2012). This study aims to extend previous findings in two ways: first, by re-examining the relationship between CI and employee exhaustion and the mediating role of SA in this relationship, while controlling for employees’ trait positive and trait negative affectivity (NA: Study 1); and second, by investigating whether employees’ trait emotional intelligence (EI) moderates the positive relationship between CI and employee exhaustion, and the link between CI and SA (Study 2).

## Customer Incivility and Its Effect on Employee Burnout

Customer incivility, which refers to “low-intensity deviant behavior perpetrated by someone in a customer or client role, with ambiguous intent to harm an employee, in violation of social norms of mutual respect and courtesy” (Sliter et al., 2010, p. 468), is a widespread phenomenon in the service industry (Andersson and Pearson, 1999; Kern and Grandey, 2009; Wilson and Holmvall, 2013; Sliter and Jones, 2016; Sommovigo et al., 2019b). CI covers customer behaviors that are rude and discourteous, thereby exhibiting disrespect for other people (Andersson and Pearson, 1999; Pearson et al., 2001; Wilson and Holmvall, 2013; Sliter and Jones, 2016). Examples of uncivil customer behaviors include ignoring employees and making disrespectful comments about them, blaming employees for problems they did not cause, and making negative comments about the organization (Wilson and Holmvall, 2013).

Although most workplace incivility research focuses on interactions between co-workers (Cortina et al., 2001; Reich and Hershcovis, 2015), there is evidence to suggest that CI is more frequent, meaning that employees are more likely to experience uncivil behaviors from customers than from co-workers (Grandey et al., 2007; Sliter et al., 2012). This may be due to several reasons. First, customer service representatives interact with customers more often than with co-workers and supervisors (Dormann and Zapf, 2004). Second, unlike interactions with co-workers, interactions with customers are usually of short duration, with limited prior history, and with little expectation of future interaction (Gutek et al., 1999). Therefore, the customer-employee relationship guarantees customers a certain level of anonymity, thereby increasing the probability of uncivil behavior on their part (Grandey et al., 2007). In contrast, organizational members must take into account possible negative consequences (i.e., formal reprimands or sanctions) of expressing uncivil behaviors toward each other or their customers (Grandey et al., 2007). Third, while the members of an organization are usually equal in status, customers and employees are not (Ben-Zur and Yagil, 2005). The employee wants the customer to buy the product or service, and the customer decides whether the transaction will ultimately take place; thereby, it is the customer who has the power in this relationship. Finally, it is the customer who is asked if s/he is satisfied with the course of the interaction with the employee and not the other way around.

Customer incivility can be considered as a specific category of daily hassles (Zohar, 1997; Cortina et al., 2001; Sliter et al., 2010). A daily hassle is a term used in the stress research literature that refers to minor everyday episodes, encounters, and/or experiences that constitute a source of annoyance, frustration and irritation for an individual (Lazarus and Folkman, 1984). When daily hassles are experienced continuously and/or in great amounts, they become a considerable source of stress (Lazarus and Folkman, 1984; Zohar, 1997). This occurs because additional energy is needed to overcome daily hassles, beyond the energy used to achieve a goal and perform a job task. Dealing with rude, overly loud and complaining customers can be regarded as a daily work hassle that evokes negative emotions in employees and makes everyday tasks more difficult and demanding than anticipated, thereby leading to strain and stress (Kanner et al., 1981). This claim is consistent with the Affective Events Theory (AET; Weiss and Cropanzano, 1996), which argues that the work environment is saturated with affective events or episodes, which are the direct cause of employees’ affective reactions, which in turn influence their behavior, attitudes and well-being (Connolly and Viswesvaran, 2000; Brief and Weiss, 2002).

Building on AET (Weiss and Cropanzano, 1996), one can assume that uncivil customer behaviors evoke negative emotions in employees, which ultimately lead to a deterioration of their well-being. Indeed, a recently published experimental study demonstrated that participants exposed to CI reported more negative emotions than their counterparts in a control condition (Sommovigo et al., 2020; see also Rupp and Spencer, 2006). There is also evidence that experiencing negative emotions increases one’s level of physiological and psychological arousal, which, cumulatively, has a harmful effect on affective and cognitive functioning (e.g., Schröder, 1995; Szczygieł et al., 2012), mental and physical health (Lazarus and Cohen-Charash, 2001; Gross et al., 2011), and contributes to employee burnout (Wright and Cropanzano, 1998; Grandey et al., 2004; Szczygieł and Mikolajczak, 2018).

The adverse effect of CI on employee well-being can be seen across various service industry environments. Research conducted among retail employees demonstrated that CI was positively associated with stress appraisal (Kern and Grandey, 2009) and emotional exhaustion (Kern and Grandey, 2009; Hur et al., 2015). Sliter et al. (2010) reported that CI correlated positively with emotional exhaustion and negatively with customer service quality in a sample of bank tellers. Likewise, in a sample of retail and restaurant student employees, Wilson and Holmvall (2013) demonstrated that an increase in employees’ perceived CI had a significant positive effect on their general psychological stress and job-specific strain. Furthermore, Sommovigo et al. (2019a) reported a positive correlation between CI and burnout among psychology students working in retail sales and restaurant services. A similar positive association between CI and employee distress was demonstrated in a sample of university alumni employed in various professions related to services, such as education, social services and health care, as well as in a sample of employees working in an engineering firm (Adams and Webster, 2012). Han et al. (2016) observed that CI resulted in restaurant frontline service employee burnout and turnover intention. The positive relationship between CI and employee emotional exhaustion was corroborated in a study conducted among customer service representatives employed in a call center (van Jaarsveld et al., 2010) and among restaurant frontline service employees (Cho et al., 2016).

According to incivility spiral effect of Andersson and Pearson (1999), employees frequently exposed to rude and disrespectful customer behaviors are more likely to retaliate against customers (van Jaarsveld et al., 2010; Walker et al., 2014, 2017), which may in turn reduce organizational performance and increases customer turnover, ultimately leading to revenue losses (Walker et al., 2014).

The above studies indicate that dealing with rude customers is costly for both individuals and organizations. The situation of service employees is further complicated by the fact that they do not have much freedom in expressing felt emotions, especially negative ones. Grandey’s (2000) emotional labor model expands and complements the predictions of AET, suggesting that not merely the emotions experienced by service workers, but the regulatory effort they make to fake and/or hide them explains how uncivil customer behaviors translate into the worsening of employee well-being (see also Grandey and Gabriel, 2015; Grandey and Melloy, 2017).

## The Role of Surface Acting in the Relationship Between Customer Incivility and Employee Burnout

Hochschild (1983) introduced the term “emotional labor” to describe the process of managing affective displays in a customer service context in order to comply with organizational display rules. Hochschild (1983) differentiates between two forms of emotional labor: SA and DA (see also Grandey, 2000; Scott and Barnes, 2011). SA is a modification of one’s own emotional manifestations without changing one’s inner feelings; DA refers to the modification of actual feelings in order to evoke an appropriate emotional display. Grandey (2000) situated the concepts of deep and SA within the framework of Gross’s emotion regulation theory (Gross, 1998, 2013) and proposed that DA corresponds to antecedent-focused emotion regulation, the aim of which is to change the situation or cognition in order to manage feelings, whereas SA corresponds to response-focused emotion regulation, the aim of which is to change expression and behavioral responses after an emotion has been felt and response tendencies have been activated (Grandey, 2000; Grandey and Gabriel, 2015; Szczygieł and Baryła, 2019). Deep and SA, defined as emotion regulation strategies, became the focal point of Grandey’s (2000) model of emotional labor.

Grandey’s (2000) model suggests that emotion regulation acts as a mechanism indirectly linking negative events at work, such as negative interactions with customers, to employee strain and psychological distress. First, Grandey’s (2000) model proposes that negative events at work have an impact on the amount of emotional labor that an employee has to perform. The reason for this is that if an event gives rise to emotions that are contrary to the emotions prescribed by the organization, then the employee has to engage in emotional labor in order to meet the requirements of the job. Second, Grandey’s (2000) model posits that engagement in emotional labor strategies consumes a considerable amount of an employee’s psychological resources (Grandey and Gabriel, 2015) and, thus, it results in psychological strain and distress. It should be emphasized that evidence constantly shows that the negative consequences of emotional labor, such as psychological stress, psychosomatic complaints and burnout symptoms, are mainly related to its surface form (Bono and Vey, 2005; Hülsheger and Schewe, 2011; Kammeyer-Mueller et al., 2013; Grandey and Gabriel, 2015; Huppertz et al., 2020). Huppertz et al. (2020) demonstrated that the main mechanisms through which SA leads to increased employee strain and burnout are: regulatory effort and a sense of unauthenticity. Moreover, research shows that employees using more SA are more likely to be mistreated by customers, which in turn increases their negative emotions and exhaustion (Zhan et al., 2016).

The assumption that SA mediates the relationship between CI and service employee well-being has been examined in a few studies. Hur et al. (2015) observed that CI was linked to SA, while SA was positively related to emotional exhaustion in a sample of retail employees. Adams and Webster (2012, Study 1) identified that SA mediated the relationship between CI and psychological distress in a sample of university alumni working in the service industry. Similar results were obtained in a study conducted by Sliter et al. (2010), who demonstrated that suppressing negative emotions and faking positive emotions fully mediated the relationship between CI and emotional exhaustion in a sample of bank tellers. Adams and Webster (2012) considered both emotional labor strategies as potential mediators in the relationship between CI and service employee occupational stress (Study 2). The results of their work demonstrated that while SA mediated the relationship between CI and employee distress, DA did not show such an effect.

It should be noted, however, that in all of the above-mentioned studies employee dispositional affectivity (or trait affectivity; Watson et al., 1988) was not controlled for, which limits the interpretation of the findings. Not including/controlling for dispositional affectivity in research is striking because there is significant evidence to show that dispositional affectivity (Watson et al., 1988) is associated with all the variables that are considered here. First, across studies, negative affectivity (NA) is related to higher levels of SA (Bono and Vey, 2005; Hülsheger and Schewe, 2011; Mesmer-Magnus et al., 2012; Kammeyer-Mueller et al., 2013). Second, there is ample evidence showing that employees higher in NA tend to report more psychological stress and burnout symptoms, while those higher in positive affectivity (PA) tend to report less stress and burnout symptoms (Zohar, 1997; Wright and Cropanzano, 1998; Grandey et al., 2004). Third, research shows that perceived CI is positively related to NA and negatively related to PA (Shin and Hur, 2019; Sommovigo et al., 2019a). Similar results were obtained by Sliter and Jones (2016), who observed a significant and positive correlation between employee neuroticism (which roughly corresponds to NA) and CI. Therefore, the correlation between CI and SA, as well as the correlation between SA and burnout scores, may be simply the result of their associations with employee dispositional affectivity (these concerns have also been raised by Bono and Vey, 2005 and Sliter et al., 2010).

In light of the concerns that CI and SA may be only spuriously associated with burnout, and that the actual “driver” of these relationships is employee trait affectivity, this study aims to contribute to the literature by investigating whether SA has a mediating effect on the relationship between CI and employee exhaustion beyond the positive and negative trait affectivity of employees. Thus, we propose the following:

*Hypothesis 1*: Surface acting mediates the positive relationship between customer incivility and employee exhaustion while controlling for employees’ positive and negative trait affectivity.

We use the term “exhaustion” rather than “emotional exhaustion” for two reasons. First, we understand exhaustion as a concept referring not only to energy loss and feelings of being emotionally drained by one’s work, but also to physical fatigue and cognitive weariness (see Schaufeli et al., 2009). Second, we use the Oldenburg Burnout Inventory (see section “Materials and Methods”), which measures the exhaustion dimension of burnout (rather than emotional exhaustion).

There is another issue that we believe should be addressed. Although CI may have an important effect on employee exhaustion, it seems unlikely to affect all employees in a similar way. Therefore, the following questions arise: Does CI *always* lead to employee exhaustion? Does CI *always* increase the use of SA? In order to answer these questions, we will refer to the Job-Demands Resources theory (JD-R; Demerouti et al., 2001; Bakker and Demerouti, 2017) and Grandey’s emotional labor model (Grandey, 2000; Grandey and Melloy, 2017) as a theoretical framework, and examine whether emotional intelligence (EI) mitigates (buffers) the negative effect of CI on employee exhaustion.

## The Moderating Role of Emotional Intelligence in the Relationship Between Customer Incivility and Burnout

According to JD-R, the characteristics of a job can be broadly classified into two groups: job demands and job resources. Job demands refer to those aspects of the job that require constant physical and/or psychological effort from employees and are, therefore, linked to certain physiological and/or psychological costs, while job resources are related to job characteristics that are functional in achieving work goals and that promote personal growth and development (Demerouti et al., 2001). In the early phase of research in JD-R, scholars emphasized the favorable role of organizational resources (e.g., social support). Later, research focused more on personal resources (Xanthopolou et al., 2007; Bakker and Demerouti, 2017). In this study, we focus on EI, an individual resource that is particularly relevant to the issues addressed here and that has drawn much scientific attention in organizational settings over the past decades (Côté, 2005; Dahling and Johnson, 2013; Lopes, 2016).

Since the notion of EI was introduced into the scientific literature (Salovey and Mayer, 1990), a number of different EI conceptualisations have been developed, which can be classified into two groups: ability models (e.g., Mayer and Salovey, 1997) and trait models (e.g., Petrides and Furnham, 2003). Ability EI (assessed by performance tests referring to maximum performance) is defined as the ability to identify, understand, regulate and utilize one’s own and other people’s emotions (Mayer and Salovey, 1997). Trait EI (assessed by self-report instruments referring to typical performance) is defined as a lower order personality trait relating to a set of emotion-related dispositions (Petrides et al., 2007). Thus, the former refers to ability to use emotions and emotional knowledge (i.e., what an individual is capable of doing in an emotionally charged situation), while the latter refers to people’s self-perceptions of their emotional abilities, as well as their self-confidence and belief in these abilities (“emotional self-efficacy,” Petrides et al., 2007). In the present study, we refer to trait EI, we are concerned about what a person is actually doing in real-life situations (i.e., how many of his or her abilities reveal themselves in emotionally charged situations).

Recent meta-analyses demonstrate that trait EI predicts positive outcomes, such as better health (Martins et al., 2010; Sarrionandia and Mikolajczak, 2019), greater sense of well-being (Andrei et al., 2016; Sánchez-Álvarez et al., 2016), better job performance (O’Boyle et al., 2011), higher job satisfaction, higher organizational commitment, and lower turnover intentions (Miao et al., 2017). There is also evidence that trait EI acts as a protective factor against the adverse effects of stressors (for a review, see Lea et al., 2019). For example, Mikolajczak et al. (2009) observed that high EI (vs. low) individuals reported a smaller increase in negative mood as a result of laboratory-induced stress. It was also demonstrated that high EI (vs. low) individuals showed significantly lower reactivity to laboratory stressors at both psychological (i.e., deterioration of mood) and physiological (i.e., salivary cortisol) levels (Mikolajczak et al., 2007a). Similar results were obtained by Laborde et al. (2014), who observed that trait EI was negatively correlated with cortisol secretion when performing tasks under pressure. Research conducted in the context of sports indicated that athletes high in trait EI exposed to a stressful stimulus (a competition-like stressor) experienced lower increase in stress (as indicated by heart rate variability) than their low in trait EI counterparts (Laborde et al., 2011). Moreover, research shows that compared to employees low in trait EI, employees high in trait EI report fewer burnout symptoms and somatic complaints, and this effect was observed in both cross-sectional (Weng et al., 2011; Szczygieł and Bazińska, 2013) as well as longitudinal studies (e.g., Mikolajczak et al., 2007b).

Building upon JD-R and trait EI theories, as well as on the above-mentioned findings, we predict that trait EI mitigates the effect of CI on employee exhaustion. Thus, we predict the following:

*Hypothesis 2*: The relation between customer incivility and exhaustion is stronger among employees low in trait EI and weaker among employees high in trait EI.

Grandey and Melloy (2017) suggested that trait EI can weaken the link between negative events and SA. Indeed, research shows that high EI individuals are less likely to report using SA (Austin et al., 2008; Mesmer-Magnus et al., 2012). Giardini and Frese (2006) found that trait EI lessens the positive relationship between emotional demands and emotional dissonance (i.e., the discrepancy between organisationally prescribed emotions and genuinely felt ones; Bono and Vey, 2005). How can this beneficial effect of trait EI be explained?

Petrides and Furnham (2003) observed that high EI (vs low) individuals are more sensitive to emotionally laden stimuli. This suggests that emotionally intelligent individuals are more likely to pay attention to their negative emotions and, thus, can act more quickly to change unpleasant affective states, namely, early on in the emotion generation process, before emotional response tendencies become fully triggered, which is likely to protect them from using SA (Gross, 1998, 2013; Grandey, 2000). Indeed, there is evidence showing that when faced with stressful situations, high EI (vs. low) individuals, are more likely to view stressful situations as a challenge, not a threat (Mikolajczak and Luminet, 2008) and to use more adaptive strategies for regulating their emotions (for a review of the most robust studies on this issue, see Peña-Sarrionandia et al., 2015).

Accordingly, we suggest that high (vs. low) trait EI individuals are better equipped to deal with stressful customer interactions, which results in less negative emotions and, consequently, less use of SA. Thus, we propose:

*Hypothesis 3*: The relation between customer incivility and surface acting is stronger among employees low in trait EI and weaker among employees high in trait EI.

**Figure 1:**
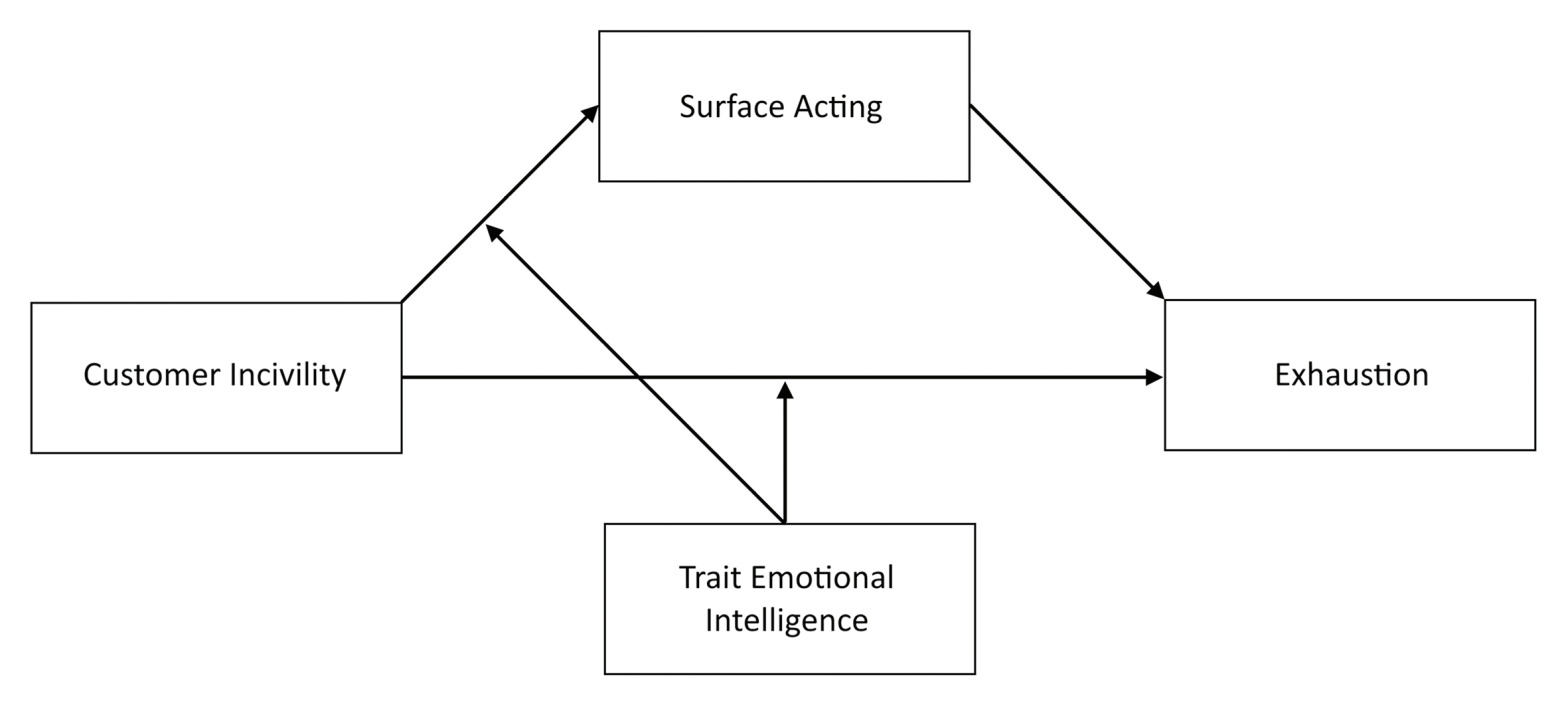
Proposed moderated mediation model in which the effect of customer incivility on exhaustion is moderated by emotional intelligence (EI). Surface acting is the proposed mediator of the conditional effect of customer incivility on exhaustion.

In summary, as displayed in Figure 1, we hypothesize that trait EI buffers both the relationship between CI and exhaustion (H2) and between CI and SA (H3). Therefore, we predict that the indirect effect of CI on exhaustion, through SA, will be contingent on employees’ trait EI. Consequently, we posit the following overall hypothesis:

*Hypothesis 4*: Trait EI moderates the positive direct and indirect effect of customer incivility on exhaustion (through surface acting). Specifically, the indirect effect of customer incivility on exhaustion through SA depends on employee EI in such a way that the relationship is weaker for employees high in EI and is stronger for employees low in EI.

## Empirical Studies

In this section, we present two empirical studies. In Study 1, we sought to examine whether CI affects employee exhaustion through its effect on SA, while controlling for employees’ trait positive and trait negative affectivity (Hypothesis 1). In Study 2, we sought to replicate the results of Study 1 and extend it by testing a moderated-mediation model in which the magnitude of the direct and indirect (through SA) relationship between CI and employee exhaustion depends on the trait EI of employees (Hypotheses 2–4).

## Analytical Procedure

Data were analyzed using SPSS 25.0. First, the descriptive statistics (i.e., means and standard deviations) and bivariate correlations among all key variables were computed. Then, we ran the set of mediation and moderated mediation analyses using Hayes’ PROCESS macro 3.2. for SPSS (Hayes, 2018). The PROCESS macro provides point estimates, and their bias and accelerated confidence intervals (BCa CIs) for all effects. BCa CIs that exclude zero suggest a significant effect. A bootstrapping procedure based on 5,000 bootstrapped resamples was used to estimate BCa 95% CIs for the effects, according to Hayes (2018).

In order to test Hypothesis 1 (Study 1) predicting that SA would mediate the relationship between CI and employee exhaustion while controlling NA and PA, we used a simple mediation model (Model 4 from PROCESS macro). In order to examine Hypotheses 2, 3, and 4 (Study 2) on the moderating role of trait EI in the relationship between CI and exhaustion mediated by SA, we conducted a moderated mediation analysis (Model 8 from PROCESS macro) testing the moderation of both the direct and indirect paths. The predicted model is shown in Figure 1. Prior to the analysis, the predictors were mean-centred. Both models (Studies 1 and 2) were controlled for intensity of customer contact and socio-demographic variables (age, gender: 1 = females, 2 = males). As recommended by Hayes (2018), unstandardized regression coefficients were reported.

## Study 1

### Materials and Methods

#### Participants

A total of 315 retail sales employees (i.e., customer service assistants and cashiers) working in shopping centres located in northern Poland (Pomeranian District) participated in this study. The criteria for inclusion in this study were as follows: voluntary participation; working in their current position in retail sales for at least 6 months; having direct contact with customers (face-to-face) for at least 50% of the working day. The exclusion criteria were: unwillingness to participate in this study (12 persons refused to participate); returning incomplete questionnaires. A total of 354 individuals expressed an initial interest in taking part in this study, of which 315 ultimately participated (89%). Thirty-nine participants were excluded from the final sample because they either did not complete fully the questionnaires (17 individuals) or were not present at the time of collection (22 individuals). The final sample had a greater number of female respondents (62.20%) than male respondents (37.80%). The participants were on average around 30 years of age with average job tenure of 8.12 years (*SD* = 5.38). Their average tenure with their current employer was approximately 6.5 years (*M* = 6.44 years, *SD* = 5.10) and ranged from 1 to 19 years. Of all the respondents, 39.4% reported being graduates of vocational or high schools, whereas 60.6% reported that they had a university degree. The participants declared spending on average 80% of their time on the job in direct (face-to-face) contact with customers.

#### Measures

##### Customer Incivility

CI was measured using the Incivility from Customer Scale developed by Wilson and Holmvall (ICS; 2013). As the original items of the ICS were developed in English, they were subjected to a forward-and back-translation process. A similar solution was adopted by researchers using this scale in Korea (Shin and Hur, 2019) and Italy (Sommovigo et al., 2019a). First, the original items were translated into Polish by the first author of this study. Next, the forward translation was reviewed by a bilingual psychologist who, after introducing a few minor changes, accepted the translation. Next, the modified version of the questionnaire was back-translated into English by an independent translator. The back-translation was found to be highly consistent with the original version of the ICS. Eventually, the final version of the questionnaire was discussed with 11 pre-test respondents, who were psychology students, working part-time as shop assistants in department stores and who were, thus, representative of the study participants. This process resulted in the Polish version of the ICS that was used in this study. The ICS consists of 10 items that inquire about the frequency of experienced uncivil customer behaviors. Participants were asked to indicate how often, over the past month, they had been confronted with rude customer behavior in their current workplace. Items were scored on a seven-point rating scale, ranging from one (never) to seven (more than three times per day). For example, participants were asked how often customers have “made gestures (e.g., eye rolling and sighing) to express their impatience,” “made negative remarks to you about your organization,” “blamed you for a problem you did not cause”. Scores for the ICS were calculated by averaging the responses to the items.

##### Surface Acting

SA was measured using five items from the Emotional Labor Scale (ELS), developed and validated on the Polish population by Bazińska et al. (2010). Examples of the items are “I do not really feel the emotions I present to customers,” “I show feelings that are different from what I feel inside”. Participants were asked to answer items in response to the question: “In order to do your job effectively on an average day at work, how often do you do each of the following when interacting with customers?” Items are scored on a seven-point rating scale, ranging from one (seldom) to seven (always). Scores were calculated by averaging the responses to the items.

##### Exhaustion

Exhaustion was measured with the eight-item exhaustion subscale of the Oldenburg Burnout Inventory (OLBI; Demerouti et al., 2003, 2010; Polish version by Baka and Basińska, 2016). Examples of the items are: “There are days when I feel tired before I arrive at work,” “During my work, I often feel emotionally drained”. Items are scored on a four-point rating scale, ranging from one (strongly agree) to four (strongly disagree). Scores were calculated by averaging the responses to the items, after appropriate items were reversed.

##### Dispositional Affectivity

Trait negative and positive affectivity were assessed using the Positive Affectivity Negative Affectivity Schedule (PANAS, Watson et al., 1988; Polish version by Brzozowski, 2010). PANAS is a 20-item scale comprising 10 negative (e.g., irritable) and 10 positive (e.g., happy) adjectives that describe emotional states. Participants were asked: “To what extent do you generally feel this way, on average, across all situations?” Items were scored on a five-point rating scale, ranging from one (very slightly or not at all) to five (extremely). Scores for the scale of negative and positive affectivity were calculated by averaging the responses to the appropriate items.

##### Procedure

Participants were recruited by psychology students who volunteered to participate in this project. First, the purpose of the study (i.e., an assessment of occupational stress in service occupations) was explained to the store managers. All store managers agreed that employees could be invited to participate in the study but the majority did not allow the study (i.e., filling out questionnaires) to take place during working hours. It was, therefore, agreed that the participants would be asked to complete the questionnaires at the end of their working day. Next, participants were personally (face-to-face) asked to participate in the study, and were informed about its purpose and the voluntary nature of participation. Employees who gave informed consent to participate began by filling out questionnaires on demographic data and trait affectivity. In order to ensure the anonymity of the study, participants were asked to create their own “pseudo-code.” They received an envelope containing questionnaires on CI and emotional labor and were asked to complete these instruments over the course of a few days, at the end of their shift. The sealed envelopes were collected from the participants 7–10 days later by the same psychology student who initiated the study. On the day the envelopes were collected, the participants completed the burnout inventory. This procedure applied to all participants. They were also assured that the data collected would be kept confidential and would only be used for research purposes. Participants did not receive any compensation for participation in the study. Data were collected from the beginning of September until mid-December 2018. All subjects gave written informed consent in accordance with the Helsinki Declaration.

## Results

### Preliminary Results

Table 1 contains the means, standard deviations, internal consistency coefficients (Cronbach’s a) and intercorrelations of the variables measured. The pattern of bivariate correlations between the variables was in line with our expectations. CI was significantly and positively associated with exhaustion and SA. NA was positively correlated with exhaustion, CI and SA. In contrast, PA was negatively correlated with exhaustion, CI and SA. Intensity of customer contact (i.e., customer contacts/day) was positively related to burnout and SA. Younger employees reported spending more time with customers and using more SA. Female participants reported a higher score on NA than did male participants, *t*(313) = 2.68, *p* < 0.01, *M* = 1.83 (*SD* = 0.57) and *M* = 1.66 (*SD* = 0.55) respectively. Furthermore, compared to male participants, female participants reported spending more time with customers, *t*(313) = 2.42, *p* < 0.01, *M* = 81.59 (*SD* = 11.86) and *M* = 78.15 (*SD* = 12.85) respectively.

**Table 1:** Study 1 means, standard deviations, intercorrelations, and internal-consistency reliability (Cronbach’s *α*) of study variables.

Variable	*M*	*SD*	*α*	1	2	3	4	5	6
1. Exhaustion	2.50	0.60	0.76	−					
2. Customer incivility	2.78	1.02	0.95	0.30^***^	−				
3. Surface acting	3.44	1.48	0.84	0.46^***^	0.29^***^	−			
4. Trait negative affectivity	1.77	0.58	0.84	0.38^***^	0.24^***^	0.28^***^	−		
5. Trait positive affectivity	3.44	0.72	0.90	−0.28^***^	−0.22^***^	−0.12^*^	−0.24^***^	−	
6. Customer contact/day (%)	80.29	12.34	−	0.07	0.02	0.17^**^	0.04	0.01	−
7. Age	29.97	5.40	−	−0.06	0.01	−0.14^*^	−0.08	−0.08	−0.11^*^

* *p* < 0.05;

** *p* < 0.01;

*** *p* < 0.001 (all two-tailed significance tests).

### Mediation Analysis

We tested the mediation model with CI as the independent variable and exhaustion as the dependent variable. In order to rule out the possibility that associations between the variables are the result of employee dispositional affectivity, NA and PA were included as the covariates. Furthermore, given that previous research suggests that employee age and gender, as well as the intensity of customer contact may be associated with SA and burnout symptoms (Purvanova and Muros, 2010; Scott and Barnes, 2011), we included these variables into the set of covariates.

The results, as displayed in Table 2, showed that more CI is related to higher SA (a/*B* = 0.322, *p* < 0.001), which in turn was associated with higher employee exhaustion (b/*B* = 0.136, *p* < 0.001). The direct effect, however, remained significant (c’/*B* = 0.065, *p* = 0.026) showing that SA only partially mediated the relationship between CI and employee exhaustion (indirect effect = 0.044, 95% CI = 0.02–0.071). NA and PA as the covariates were found to be significant, but none of the socio-demographic variables (age and gender) and the intensity of customer contact effects was significant. These results support our H1 by demonstrating the indirect effect of CI on burnout through the mediation of SA, beyond employee negative and positive affectivity.

**Table 2:** Coefficients for the tested mediation model.

Predictors		Outcome *M*: Surface acting					Outcome *Y:* Exhaustion			
		Coeff.	SE	LLCI	ULCI		Coeff.	SE	LLCI	ULCI
Constant		1.473	0.912	−0.322	3.268		1.923	0.327	1.280	2.566
Co: Age		−0.029	0.014	−0.058	0.001		−0.001	0.005	−0.011	0.009
Co: Gender		−0.200	0.163	−0.520	0.120		0.005	0.058	−0.110	0.119
Co: Customer contacts/day		0.019^**^	0.006	0.006	0.031		0.000	0.002	−0.004	0.005
Co: Trait negative affectivity		0.558^***^	0.143	0.276	0.839		0.214^***^	0.052	0.111	0.317
Co: Trait positive affectivity		−0.069	0.111	−0.287	0.150		−0.131^**^	0.040	−0.209	−0.053
Me: Surface Acting		-	-	-	-	(b)	0.136^***^	0.020	0.096	0.176
X: Customer incivility	(a)	0.322^***^	0.0791	0.1174	0.5274	(c’)	0.065^*^	0.029	0.008	0.122
		*R*^2^ = 0.173^***^ F (6,308) = 10.706; *p* < 0.001					*R*^2^ = 0.319^***^ F (7,307) = 20.532; *p* < 0.001			
**Indirect effect**				**Boot SE**			**Boot LLCI**			**Boot ULCI**
		0.077		0.023			0.036			0.124

SE = standard error; 95% CI = confidence interval with lower and upper limits.

* *p* < 0.05;

** *p* < 0.01;

*** *p* < 0.001.

## Study 2

### Materials and Methods

#### Participants

A total of 292 customer service representatives of two mobile phone companies in Poland participated in this study. All participants worked in customer service stores servicing individual customers. The criteria for inclusion in this study were identical to those in Study 1. A total of 423 individuals initially expressed interest in this research project (out of 488 invited to participate), of which 292 (51% women) ultimately participated (69%). One hundred and thirty-one participants were excluded from the final sample because they either withdrew from the survey (42 individuals) or could not be reached due to Covid-19 Lockdown restrictions (89 individuals). The participants were on average around 36 years of age with average job tenure of 14.89 years (*SD* = 5.38). Of all the respondents, 33.2% reported being graduates of vocational or high schools, whereas 66.8% reported that they had a university degree. The participants reported spending on average 82% of their time on the job in direct (face-to-face) contact with customers.

#### Measures

In Study 2, four variables were measured: CI, SA, exhaustion and trait EI. The first three variables were measured using the same instruments used in Study 1, namely the ICS was used to measure CI, the ELS was used to measure SA, and the OLBI was used to measure exhaustion. The descriptions of these instruments can be found in Study 1. In addition to the variables listed above, in Study 2, trait EI was also measured.

#### Trait Emotional Intelligence

Trait EI was assessed with the Trait Emotional Intelligence Questionnaire-Short Form (TEIQue-SF, Petrides and Furnham, 2006; Polish version by Szczygieł et al., 2015). This questionnaire is derived from the full form of the TEIQue (for a comprehensive description of the factors and subscales, see Petrides, 2011) and contains 30 items with answers on a seven-point Likert scale ranging from 1 (completely disagree) to 7 (completely agree). Examples of items are: “Expressing my emotions with words is not a problem for me” and “I often find it difficult to see things from another person’s viewpoint” (reversed). Scores for the TEIQue-SF were calculated by averaging the responses to the items, after reversion of appropriate items.

#### Procedure

As in Study 1, the participants in Study 2 were recruited with the help of psychology students, who personally contacted employees during working hours, asking them to participate in the study. The employees were informed about the purpose of the study (i.e., the role of emotional competence in service work) and were assured of its anonymity. Employees who gave their informed consent to participate in the survey began by completing questionnaires on demographic data and CI. They were asked to create their own “pseudo-code” in order to ensure the anonymity of the study and to allow matching the questionnaires to the study participant. The employees then received an envelope with the questionnaires on emotional intelligence and emotional labor and were asked to complete them within the next few days, during their breaks. The sealed envelopes were collected from participants 5–8 days later. On the day the envelopes were collected, the participants completed the burnout inventory. This procedure applied to all participants. No compensation was offered for participation. Data was collected from mid-January to mid-March 2020 in the Masovian (central Poland) and Pomeranian (northern Poland) districts.

## Results

Descriptive statistics, internal consistency coefficients (Cronbach’s a) and intercorrelations among the variables are presented in Table 3. CI was positively associated with exhaustion, SA and intensity of customer contact. Trait EI was negatively correlated with CI, exhaustion and SA. Younger employees reported more uncivil customer behaviors. In addition, compared to female participants, male participants reported more uncivil customer behaviors, *t*(290) = 3.43, *p* < 0.01, *M* = 3.23 (*SD* = 1.03) and *M* = 2.83 (*SD* = 0.94) respectively. Furthermore, male participants declared using more SA than female participants, *t*(290) = 2.68, *p* < 0.01, *M* = 3.20 (*SD* = 1.27) and *M* = 2.82 (*SD* = 1.13) respectively. Finally, the results showed that female participants reported higher scores on trait EI than male participants, *t*(290) = 2.17. *p* < 0.05, *M* = 4.99 (*SD* = 0.47) and *M* = 4.87 (*SD* = 0.49) respectively.

**Table 3:** Study 2 means, standard deviations, intercorrelations, and internal-consistency reliability (Cronbach’s α) of study variable.

Variable	M	SD	α	1	2	3	4	5
1. Exhaustion	2.61	0.62	0.83	−				
2. Customer incivility	3.03	1.00	0.94	0.29^***^	−			
3. Surface acting	3.01	1.22	0.86	0.33^***^	0.31^***^	−		
4. Trait emotional intelligence	4.93	0.49	0.87	−0.46^***^	−0.22^***^	−0.24^***^	−	
5. Customer contact/day (%)	82.44	13.68	−	−0.03	0.12^*^	0.05	0.04	−
6. Age	35.90	7.00	−	−0.13^*^	−0.10	−0.01	0.02	−0.07

^*^p < 0.05; ^***^p < 0.001 (all two-tailed significance tests).

### Moderated Mediation Analysis

**Figure 2:**
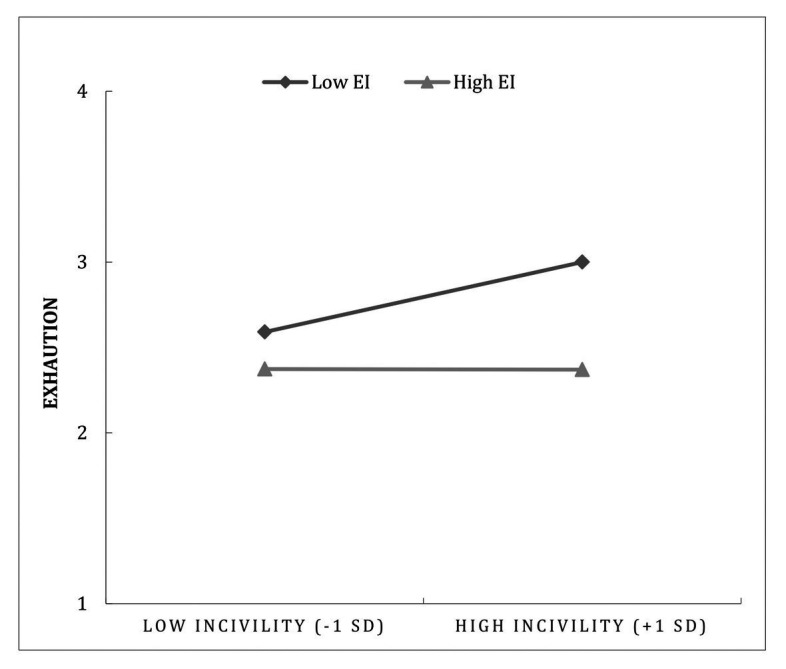
Employee’s exhaustion as a function of the interaction between customer incivility and emotional intelligence.

**Figure 3:**
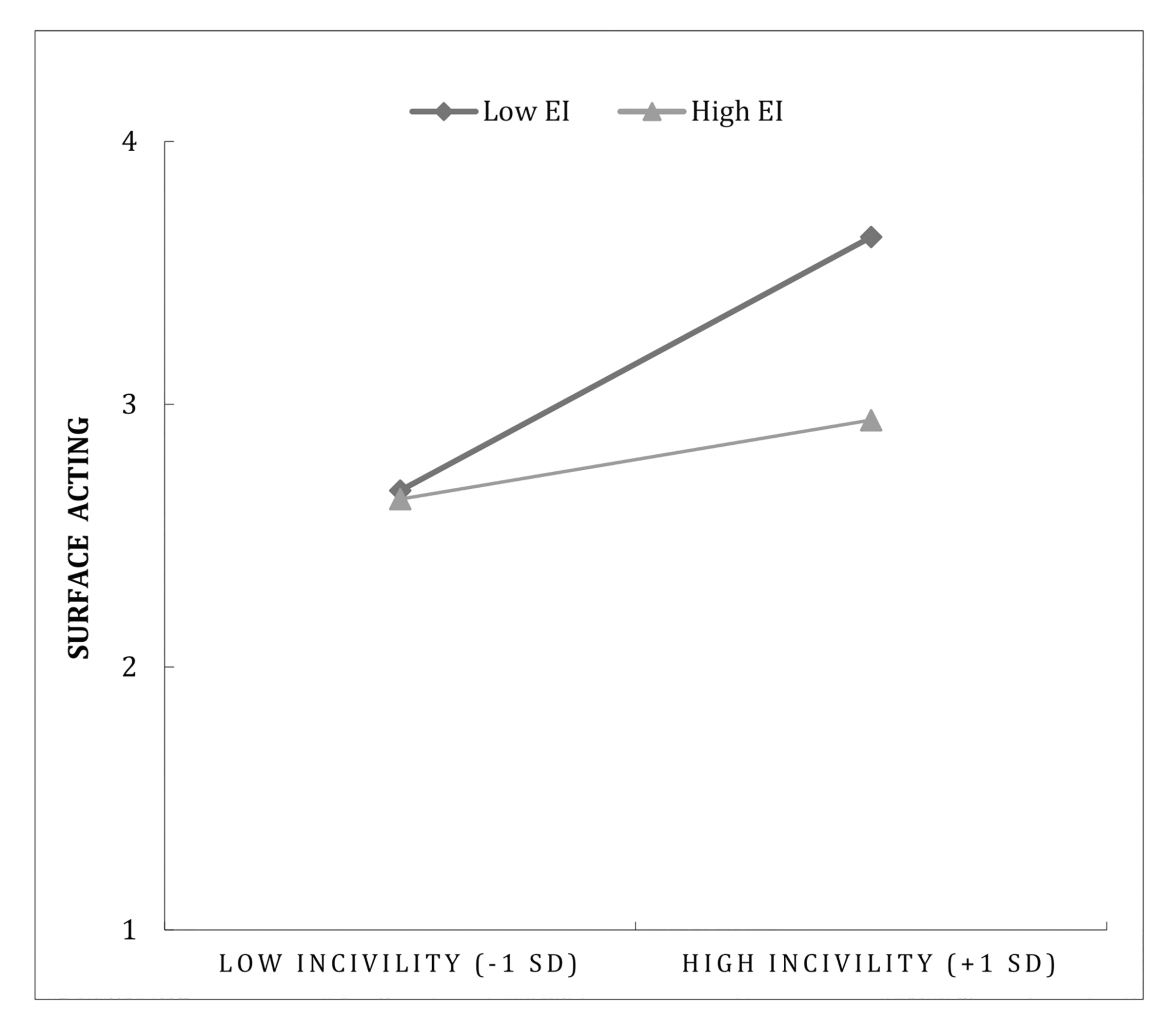
Surface acting as a function of the interaction between customer incivility and emotional intelligence.

In order to examine Hypotheses 2, 3, and 4 on the moderating role of trait EI in the relationship between CI and employee exhaustion, we conducted a moderated mediation analysis testing the moderation of both direct and indirect paths (i.e., mediated through SA). The results are presented in Table 4. In line with Hypothesis 2, the conditional direct effect (as depicted in Figure 2) was significant for participants low in trait EI [*B* = 0.482, *SE* = 0.101, *p* < 0.001, (95% CI = 0.282–0.682)] but not for participants high in trait EI [*B* = 0.146, *SE* = 0.095, *p* = 0.125, (95% CI = − 0.041–0.334)]. The index of moderated mediation did not pass through zero [*B* = − 0.030, *SE* = 0.017, (95% CI = − 0.067 to −0.003)], which indicates, in line with Hypothesis 3, that the indirect effect of CI on employee exhaustion through SA was significantly different among participants with low and high trait EI. The inspection of the conditional indirect effect indicates, in line with Hypothesis 4, that there was a significant indirect effect of CI on exhaustion through SA only among participants low in trait EI [*B* = 0.043, *SE* = 0.017, *p* < 0.001, (CI = 0.015–0.080)] but not among participants high in trait EI [*B* = 0.013, *SE* = 0.010, (CI = − 0.004–0.035)]. There was also an interaction effect between CI and trait EI on SA, as shown in Figure 3. The results showed that the conditional effect of CI on SA was significant for participants with low EI [*B* = 0.043, *SE* = 0.017, *p* < 0.001, (CI = 0.015–0.080)] but not for those with high EI [*B* = 0.013, *SE* = 0.010, (CI = − 0.004–0.035)]. In sum, these results support Hypotheses 2, 3, and 4.

**Table 4:** Coefficients for the tested moderated mediation model.

Variables	Outcome *M*: Surface acting				Outcome *Y*: Exhaustion			
	Coeff.	SE	LLCI	ULCI	Coeff.	SE	LLCI	ULCI
Constant	2.783	0.592	1.617	3.949	2.760	0.281	2.208	3.312
X: Customer incivility	0.314^***^	0.070	0.176	0.452	0.100^**^	0.033	0.035	0.165
Me: Surface Acting	-	-	-	-	0.088^***^	0.027	0.035	0.141
Mod: Emotional intelligence (EI)	−0.375^**^	0.144	−0.658	−0.091	−0.433^***^	0.067	−0.564	−0.302
(X × Mo) Incivility × EI	−0.344^*^	0.142	−0.623	−0.066	−0.208^**^	0.065	−0.336	−0.079
Co: Customer contacts/day	0.004	0.005	−0.006	0.013	−0.002	0.002	−0.006	0.002
Co: Age	0.004	0.010	−0.014	0.023	−0.009	0.004	−0.017	0.000
Co: Gender	−0.173	0.136	−0.441	0.095	0.020	0.062	−0.103	0.143
	*R* ^2^ = 0.153 *F* (6, 285) = 8.576, *p* < 0.001				*R* ^2^ = 0.319 *F* (7, 284) = 18.990, *p* < 0.001			
**Index of moderation mediation**			**Boot SE**		**Boot LLCI**			**Boot ULCI**
−0.030			0.017		−0.067			−0.003

SE = standard error; 95% CI = confidence interval with lower and upper limits.

* *p* < 0.05;

** *p* < 0.01;

*** *p* < 0.001.

## Discussion

This study was designed to examine the effects of emotional demands in service work, namely uncivil customer behaviors and SA on employee exhaustion. Consistent with our predictions, the results of two independent studies, one of which was conducted among retail sales employees and the other among customer service representatives, demonstrated that CI significantly increased the use of SA and exhaustion.

The first objective of the current study was to re-examine the relationship between CI and employee exhaustion and the mediating role of SA in this relationship, while controlling for NA and PA. In line with previous findings (Wright and Cropanzano, 1998; Grandey et al., 2004; Hülsheger and Schewe, 2011; Kammeyer-Mueller et al., 2013; Shin and Hur, 2019; Sommovigo et al., 2019a), the results of Study 1 demonstrated that both NA and PA were significantly related to all the variables under study. In addition, it was revealed that increases in the use of SA were related to increases in exhaustion, thus providing supporting evidence for the mediating role of SA. These results are in accordance with prior research showing an indirect effect (through SA) of CI on employee exhaustion (Sliter et al., 2010; Hur et al., 2015), as well as the assumptions of Grandey’s (2000) emotional labor model. Importantly, the mediating effect of SA in the CI–exhaustion relationship emerged while controlling for employee affectivity, which supports our Hypothesis 1. These are important findings, as they rule out the possibility that the relationships between the variables examined here are simply a function of employee dispositional affectivity and its effects on the rest of the variables.

The second objective of this research was to examine whether CI *always* leads to an increased use of SA and ultimately to increased employee exhaustion. The results of Study 2 support a moderated mediation model in which trait EI buffers the direct and indirect (through SA) effects of CI on exhaustion. Specifically, it was found that employees exposed to many uncivil customer behaviors but high in trait EI reported using less SA and, thus, experienced fewer exhaustion symptoms than their low in trait EI counterparts. The highest scores on SA and exhaustion were reported by employees low in trait EI. These results provide support for our Hypotheses 2 and 3 and suggest that in order to avoid/minimize personal costs resulting from interactions with rude and disrespectful customers; employees must have a particular personal resource at their disposal, namely EI. In other words, employees have to be able to deal effectively with emotionally charged situations (Petrides, 2011). This conclusion is supported by the results of earlier studies demonstrating that trait EI mitigates the effects of negative emotions felt at work on burnout (Szczygieł and Mikolajczak, 2018), as well as the effects of interpersonal conflicts at work on emotional exhaustion (Szczygieł and Baka, 2016).

Our research contributes to the literature on customer mistreatment in three ways. First, it responds to calls for studies that include EI in predictions of workplace behavior (Lopes, 2016), especially in jobs with high emotional labor requirements (Dahling and Johnson, 2013). Second, it highlights EI as important personal resources in service work and, therefore, bears some practical implications. Given that emotionally demanding interactions with customers seem to be an inevitable part of service work, organizations may want to consider providing EI training programmes for their employees to help them increase their emotional skills. Trait EI represents a relatively stable disposition, but recent findings are optimistic in their indication that EI training focusing on basic emotional competencies, such as understanding, regulation and the use of emotions, is effective even within a relatively short time span. For an overview of the most robust studies on this issue, see Kotsou et al. (2018) and for a meta-analysis, see Mattingly and Kraiger (2019).

Third, our study was conducted in Poland, thus responding to a call to extend workplace incivility research to a greater number of countries (Schilpzand et al., 2016). This is important, as most research in this area has been conducted in developed countries, whose economies have been dominated by the service industry for decades (e.g., the United States, Canada and Italy). We, therefore, believe that our research is a good complement to previous studies. The relationship between customer incivility and emotional labor, and between emotional labor and burnout, therefore, seems to be similar across countries, regardless of the importance of the service sector in their economies.

It should be emphasized that our research focuses on CI, which concerns only one form of customer mistreatment. It is therefore unknown whether the effects demonstrated here would be revealed in relation to other manifestations of customer mistreatment. It is especially interesting in relation to customer aggression which is often juxtaposed with CI (Wilson and Holmvall, 2013; Sommovigo et al., 2019b). Although both CI and customer aggression violate workplace standards for treating others in the workplace and may be covered by the general term “workplace mistreatment,” they differ in terms of intensity, frequency and intentions (Wilson and Holmvall, 2013). First, incivility is ambiguous about the intention to harm the target, whereas aggression is plainly aimed at causing harm (Tepper and Henle, 2011). The intention to cause harm in an uncivil act may not be clear to the person being uncivil, the person experiencing the incivility, or the observers (Andersson and Pearson, 1999; Pearson et al., 2001; Sliter et al., 2010; Wilson and Holmvall, 2013). Second, uncivil acts are often considered less intense than aggressive behaviors (Andersson and Pearson, 1999; Wilson and Holmvall, 2013). Third, given that aggressive behaviors are more intense and intentional, they are likely to be less frequent and their impact on the target is potentially more direct than the effect of uncivil behavior (Wilson and Holmvall, 2013).

There are several limitations to the current study that suggest directions for future research. First, the cross-sectional design of the data collection precludes causal interpretations. A certain causal order of the variables was assumed, such as exhaustion resulting from the experience of CI, but other causal directions are also possible, i.e., exhausted employees may perceive customers as ruder. Future longitudinal studies might capture the reciprocal nature of these relationships well. Second, our data were based solely on self-report instruments, which could lead to concerns about common method variance (Podsakoff et al., 2003). The measured predictors, however (CI, SA, and trait EI) and the outcome variable (exhaustion) at different points in time reduced the likelihood that the results of this study were caused exclusively by common method variance. Dispositional affectivity was also controlled for (Study 1), as it constituted a more conservative test of the relationships between the variables analyzed in the study. Additional data sources, such as reports from colleagues or supervisors, as well as observational techniques, could be used in future studies to strengthen the findings. Third, we did not take into account other factors that are relevant for the relationships analyzed here. In our model we focused on the mediating role of SA in the relationship between CI and exhaustion, but research suggests that there are other potential mediators that could play an important role in this relationship (e.g., the frequency and intensity of employees’ negative and positive emotions during interactions with customers; Glomb and Tews, 2004; Zellars et al., 2004; Rupp and Spencer, 2006). There is also evidence showing that there are other factors that could moderate the relationship between CI and exhaustion. For example, research demonstrates that organizational and supervisory support mitigates the adverse effects of customer verbal aggression on emotional exhaustion among call centre workers (Li and Zhou, 2013). Likewise, sharing feelings with team members (a climate of authenticity) has been linked to reduced burnout resulting from SA (Grandey et al., 2012). There is also evidence that exposure to customer mistreatment increases cooperative and prosocial behaviors towards co-workers and customers, although this effect depends on customer orientation (Yue et al., 2017). Finally, we asked the survey participants about their experience with uncivil customer behaviors over the past month and, thus, we obtained an aggregate measure of CI. Recently, however, research increasingly moves beyond aggregated measures of CI and focuses more on specific CI encounters (event perspective) in order to gain insight into the dynamic nature of customer–employee interactions (Walker et al., 2014, 2017). Therefore, it would be advisable for further research to advance a more sophisticated research model that would capture the complexity of the potential factors (at organizational, individual, as well as service-episodic levels) that may affect the relationship between CI, SA and exhaustion more fully.

## Data Availability Statement

The datasets generated for this study are available on request to the corresponding author.

## Ethics Statement

The studies involving human participants were reviewed and approved by the SWPS University of Social Sciences and Humanities (Poland), WKE-S-19-VIII-60. The participants provided their written informed consent to participate in this study.

## Author Contributions

DS developed the study design, coordinated the data collection, and drafted the manuscript. DS and RB contributed to data interpretation. RB supervised the methodology, computed the analyses, wrote the Results section, and commented on the first draft of the manuscript. Both authors approved the final version of the manuscript.

### Conflict of Interest

The authors declare that the research was conducted in the absence of any commercial or financial relationships that could be construed as a potential conflict of interest.
